# Visual acuities in patients with autosomal recessive retinitis pigmentosa associated with four rod phototransduction genes

**DOI:** 10.1038/s41433-026-04595-x

**Published:** 2026-05-22

**Authors:** Vishanna Balbirsingh, Shaima A. Hashem, Michalis Georgiou, Siying Lin, Gavin Arno, Mariya Moosajee, Andrew R. Webster, Michel Michaelides, Omar A. Mahroo

**Affiliations:** 1 NIHR Biomedical Research Centre at Moorfields Eye Hospital and the UCL Institute of Ophthalmology, London, UK; 2 Institute of Ophthalmology, University College London, London, UK; 3 Mass Eye and Ear, Harvard Ophthalmology, Boston, MA USA; 4 Division of Evolution, Infection and Genomics, University of Manchester, Manchester, UK; 5 Manchester Centre for Genomic Medicine, Saint Mary’s Hospital, Manchester University NHS Foundation Trust, Manchester, UK; 6 Greenwood Genetic Center, Greenwood, SC USA; 7 Department of Ophthalmology, St Thomas’ Hospital, London, UK

**Keywords:** Disease genetics, Hereditary eye disease

Inherited retinal diseases (IRDs) are a leading cause of vision impairment in children and working-age adults in several countries. Retinitis pigmentosa (RP) is the most frequent phenotype. Bi-allelic pathogenic variants in any one of many genes can cause autosomal recessive RP (ARRP). Where ARRP arises from variants in rod phototransduction genes (in contrast to other genes), one hallmark is early onset nyctalopia since rod phototransduction is affected from birth, well before the onset of widespread rod photoreceptor degeneration [1]. When the latter occurs, consequent cone death in the peripheral retina leads to visual field loss. Loss of central visual acuity (VA) can occur at advanced stages when foveal cones degenerate.

Here, we compared visual acuities in ARRP associated with variants in four genes: *PDE6A* and *PDE6B* encode alpha and beta subunits of rod phosphodiesterase (PDE), which, when activated in phototransduction, hydrolyzes cyclic GMP; *CNGA1* and *CNGB1* encode alpha and beta subunits of the rod cyclic nucleotide-gated (CNG) outer segment cation channels, which allow entry of sodium and calcium ions, and which close in response to the PDE-mediated reduction in cyclic GMP. We hypothesized that the two mechanisms might lead to differing RP progression rates, which might be detectable as differences in age-adjusted VA levels. We hypothesized that with PDE dysfunction, intracellular calcium levels might be higher (CNG channels remain open due to high cyclic GMP), possibly predisposing to faster degeneration; with CNG channel impairment, by comparison, intracellular calcium levels would be likely lower (due to restricted entry).

Patients with ARRP associated with the 4 genes were identified from the electronic record of a large IRD service (Moorfields Eye Hospital) [2–4]. VA recorded at first visit was extracted and comparison made between the 2 groups (CNG vs. PDE6), followed by pairwise comparisons for the 4 genes, adjusting for age (one-way ANCOVA for two independent samples). We identified 113 patients: numbers for each gene are shown in Fig. 1A. Mean (SD) ages at first visit were 38.6 (12.5), 44.3 (14.9), 34.8 (17.4), and 36.4 (18.0) for *CNGA1*, *CNGB1*, *PDE6A* and *PDE6B* respectively (Fig. 1B). Mean logMAR acuities at first visit were 0.10, 0.34, 0.45 and 0.56, respectively. Patients with CNG-associated disease had better average VA compared with PDE-associated disease (age-adjusted *p* = 7.5 × 10^−5^; Fig. 1C). When separating by gene (Fig. 1D), pairwise age-adjusted comparisons were significant for the following: better acuities were seen for *CNGA1* compared with *PDE6A* (*p* = 8 × 10^−5^) and *PDE6B* (*p* = 0.003); better acuities were also seen for *CNGB1* compared with *PDE6B* (*p* = 0.003).

**Fig. 1:**
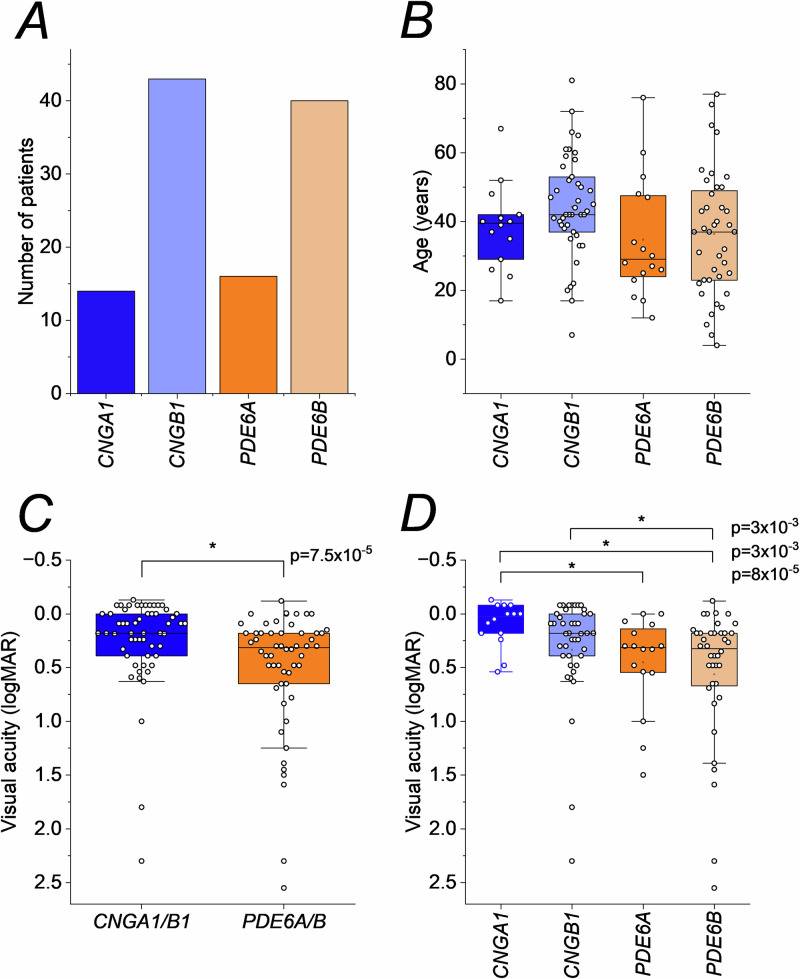
Numbers, ages, and acuities of patients with ARRP associated with selected phototransduction genes. **A** Number of patients for each gene: 113 patients in total had RP associated with *CNGA1* (*n* = 14), *CNGB1* (*n* = 43), *PDE6A* (*n* = 16) and *PDE6B* (*n* = 40). **B** Boxplots showing age at first recorded visit for the 4 genes. **C** Boxplots showing VA for patients with CNG or PDE-associated RP. The former had better VA on average (age-adjusted *p* = 7.5 × 10^−5^). **D** Boxplots showing VA by gene. Comparisons were significant for the following: better acuities were seen for *CNGA1* compared with *PDE6A* (*p* = 8 × 10^−5^) and *PDE6B* (*p* = 0.003); better acuities were also seen for *CNGB1* compared with *PDE6B* (*p* = 0.003).

Although all four RP subtypes entail congenital impairment of rod phototransduction, VA appears better preserved in variants affecting the rod CNG channels compared with those affecting rod phosphodiesterase, supporting our initial hypothesis. Such differences might arise from different effects on intracellular calcium. These data aid in understanding potential disease mechanisms and potential windows for future therapeutic intervention. Limitations of this study include relatively small numbers for some genes (limiting the power of some of the pairwise comparisons) and lack of visual field data or analysis of retinal imaging.

## Data Availability

The data processed for the analysis in this manuscript are available on request.
